# An Analog of electrically induced transparency via surface delocalized modes

**DOI:** 10.1038/srep12251

**Published:** 2015-07-21

**Authors:** Xiao Xiao, Bingpu Zhou, Xinke Wang, Jingwen He, Bo Hou, Yan Zhang, Weijia Wen

**Affiliations:** 1 Department of Physics, The Hong Kong University of Science and Technology, Clear Water Bay, Kowloon, Hong Kong; 2 Department of Physics, Capital Normal Univeristy, Beijing 100048, China; 3 College of Physics, Optoelectronics and Energy & Collaborative Innovation Center of Suzhou Nano Science and Technology, Soochow University, 1 Shizi Street, Suzhou 215006, China

## Abstract

We demonstrate theoretically and experimentally an interesting opaque state, which is based on an analog of electromagnetically induced transparency (EIT) in mechanism, in a metal hole array of the dimer lattice. By introducing a small difference to the dimer holes of each unit cell, the surface delocalized modes launching out from the dimer holes can have destructive interferences. Consequently, a narrow opaque window in the transparent background can be observed in the transmission spectrum. This surface-mode-induced opacity (SMIO) state is very sensitive to the difference of the dimer holes, which will promise various applications.

Electromagnetically induced transparency (EIT) is an interesting phenomenon for both scientific and technological aspects due to its intriguing properties such as reducing the group velocity of the light^1^ and freezing light^2^,^3^. In a well-known model the EIT state can be realized by the interference of two different excitation pathways to the upper level in a three-level atomic system^4^. Recently, a lot of proposals realizing the EIT-like phenomena in classical systems have been shown in a waveguide side coupled to resonators^5^,^6^,^7^,^8^ and the metamaterials^9^,^10^,^11^,^12^,^13^,^14^,^15^,^16^,^17^,^18^,^19^,^20^,^21^,^22^,^23^,^24^,^25^,^26^,^27^. However, all these analogs the destructive interference is achieved by the coupling of the localized resonant modes. Due to the short range characteristics of the couplings^28^,^29^,^30^, the distance between the resonant elements should be always much smaller than the wavelength^11^,^18^,^28^,^29^,^30^. Therefore, it is a natural question: can EIT-like phenomena be realized by delocalized modes, by which a clear-cut view of the interference between excitation pathways can be obtained?

In this work we consider metal hole arrays^31^,^32^,^33^ of dimer lattice. By tuning the electromagnetic property of one dimer hole slightly different from the other, the delocalized surface modes bounding on the metal surface can build up destructive interferences. This gives rise to a narrow opaque window in the transparent background in the transmission spectrum. We denote the EIT-like opaque state as the surface-mode-induced opacity (SMIO) state. Due to the delocalization of the surface modes, a clear interference pattern is shown at the SMIO state. In addition, the SMIO state is very sensitive to the electromagnetic property of the dimer holes and promises sensor applications^20^,^21^,^22^.

## Results

**Figure 1:**
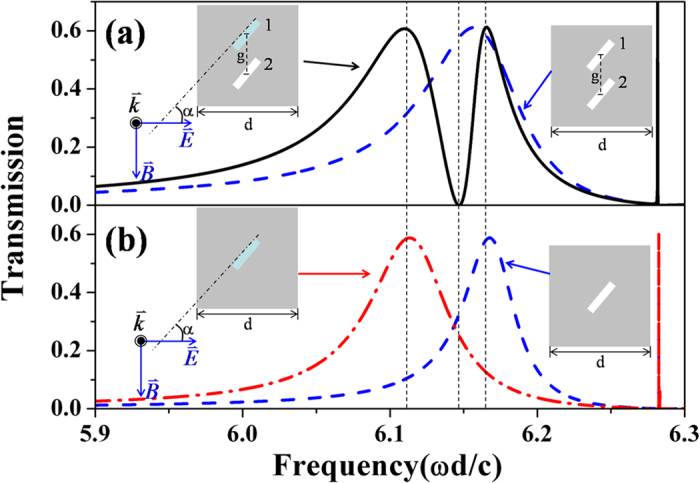
(**a**) The transmission spectra through metal hole array of dimer lattice: the black solid curve is for the case that two dimer holes in each unit cell have different dielectric constants (ε_1_ = 1.21, ε_2_ = 1); the blue dash curve is for the case that the two dimer holes have the same dielectric constant (ε_1_ = 1, ε_2_ = 1). (**b**) The transmission spectra through metal hole array of single lattice: the red dash-dot line is for the case that the dielectric constant of the hole is ε = ε_1_ = 1.21, and the blue dash line is for the case that the dielectric constant of the hole is ε = ε_1_ = 1. As it is shown in the inset, the lattice constant is ***d***, the center-center distance of the two dimer holes is ***g***** = *****0.5d***, and the long sides of the rectangular holes have an angle ***α*** = 50° from the polarization direction of the electric field. In the unit of frequency, ω is the angular frequency, c is the velocity of light in vacuum.

Metal hole array (MHA) of dimer lattice is schematically shown in the inset of Fig. 1(a). By its definition, the dimer lattice means that there are two rectangular holes in one unit cell. In the structure the longer sides of rectangular holes are parallel. The light is shining on the structure normally with the electric field polarized in the direction perpendicular to the line linking the centers of the two rectangular holes (see the inset of Fig. 1(a)). As long as the incident light can illuminate the holes, the orientation of the holes is not important^32^. Without losing generality, we assume that the longer sides tilt an angle α = 50° from the direction of the incident electric field (see the insets in Fig. 1). By using the mode expansion method^32^,^34^,^35^, we calculate the transmission spectra. As it is shown in Fig. 1(a), comparing to the transmission spectrum (the blue dash line) of MHA of identical dimer holes (ε_1_ = ε_2_ = 1), a small difference in the dielectric constants of the dimer holes (ε_1_ = 1.21, ε_2_ = 1) leads to an EIT-like spectrum (black curve in Fig. 1(a)): a sharp transmission dip is sandwiched between two transmission peaks. We find that the positions of the transmission peaks in the EIT-like spectrum are almost identical to those of the transmission peaks through single-hole arrays (Fig. 1(b)). This observation illustrates that the lower transmission peak of the EIT-like spectrum is obtained by the resonance of ε_1_ = 1.21 holes in the dimer hole array, while the peak in the higher frequency is caused by ε_2_ = 1 holes. The opaque state between the two peaks is a result of somehow hybridization of the two resonance.

**Figure 2:**
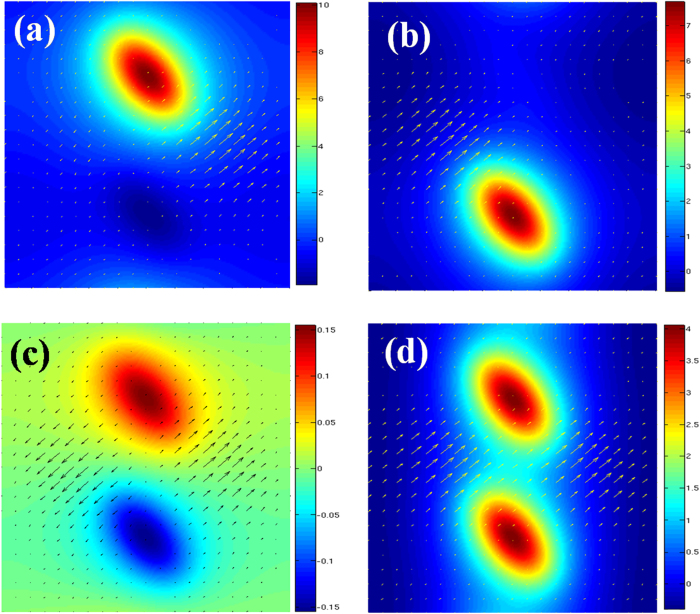
The distribution of the tangential electric field in a unit cell for different cases: **(a)** at the first transmission peak of the black solid curve in Fig. 1(a); (**b**) at the second transmission peak of the black solid curve in Fig. 1(a); (**c**) at transmission dip of the black solid curve (the SMIO state) in Fig. 1(a); (**d**) at the transmission peak of the blue dash curve in Fig. 1(a). By comparing (**c**) and (**d**), it can be seen that the opposite phase in (**c**) is crucial for the occurrence of the SMIO state.

**Figure 3:**
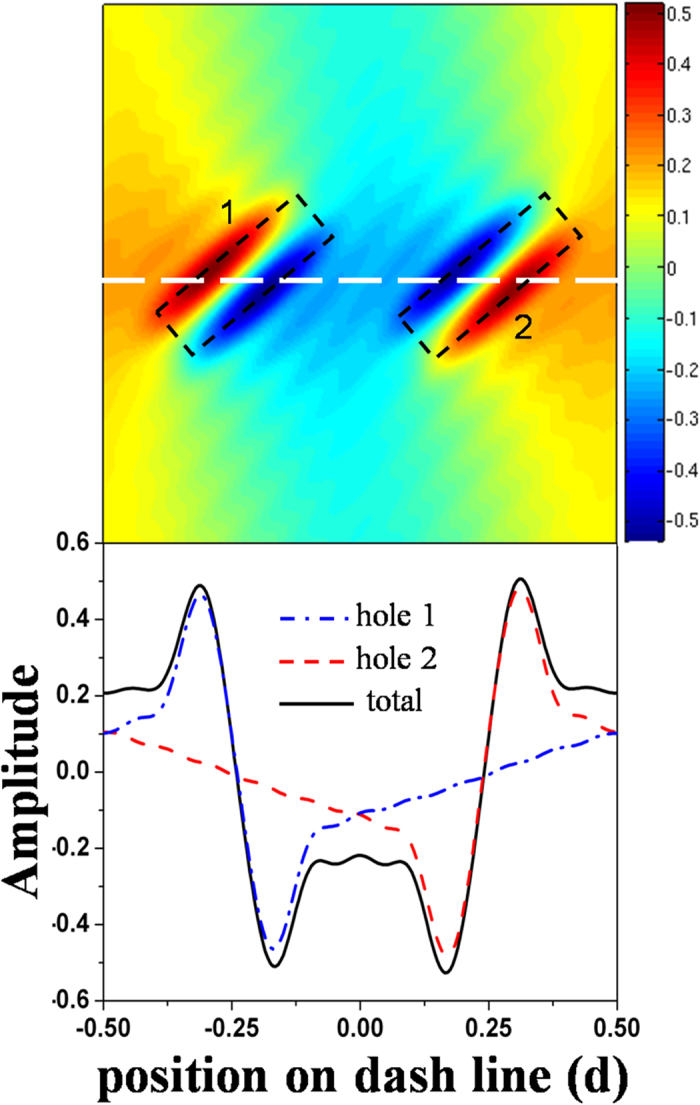
Upper panel: the distribution of surface modes (z-component of electric field) in a unit cell; the black dash boxes show the positions of the two dimer holes. **Lower panel**: the distribution of the surface modes along the white dash line highlighted in the upper panel. The blue dash-dot curve shows the distribution of the surface modes launched from the hole 1, the red dash line presents the surface modes launched from hole 2, and the black solid curve is the interference results of the surface modes from hole 1 and hole 2. Clear interference can be seen.

To confirm the above understanding, we calculate the tangential electric field (parallel to the metal surface) on the metal surface at the two transmission peaks and the opaque state. As one expected, at the lower transmission peak great field enhancement is observed around the ε_1_ = 1.21 holes (Fig. 2(a)), while the field enhancement is found around the ε_2_ = 1 holes at the higher transmission peak (Fig. 2(b)). At the opaque state, we find that both the dimer holes are at resonant (Fig. 2(c)). For a comparison, we also calculate the tangential electric field on the metal surface at the transmission peak of a MHA of identical dimer holes (ε_1_ = ε_2_ = 1) and show it in Fig. 2(d). By comparing Fig. 2 (c),(d), we find that the opposite phases at the two holes are crucial for the formation of the opaque state. To further explore the properties at the opaque state, we calculate the z-component (perpendicular to the metal surface) electric field on the metal surface. From the distribution of the z-component electric field in Fig. 3, we observe a clear interference pattern of the delocalized surface modes launched from the neighbored holes. These results clearly indicate that the opaque state is caused by the destructive interference of the delocalized surface modes.

**Figure 4:**
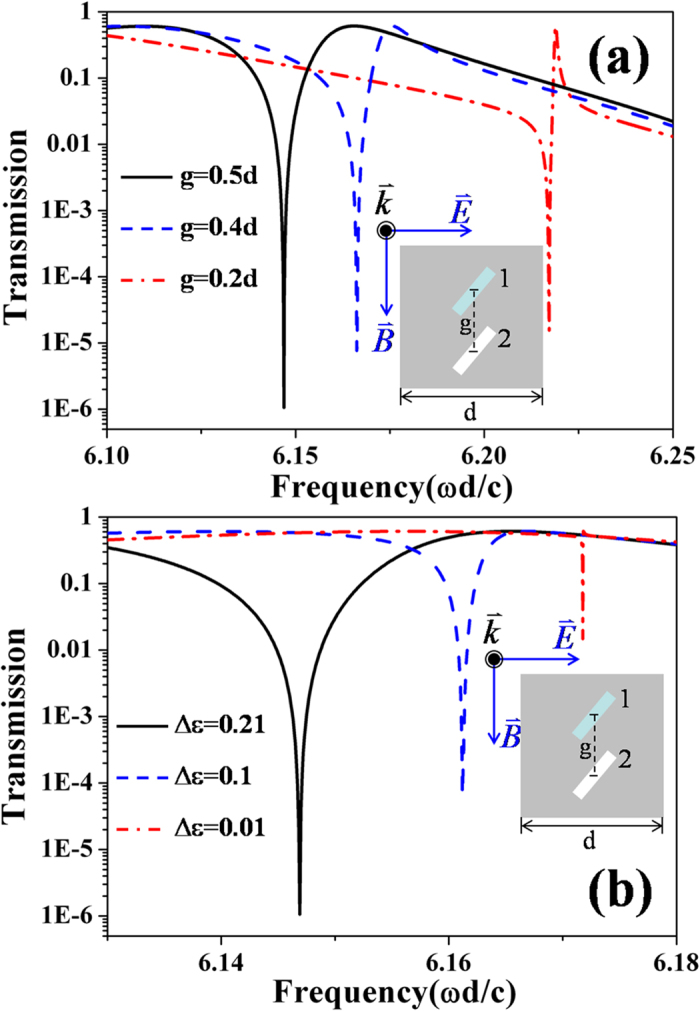
(**a**) The dependence of the SMIO state on the center-center distance of the two dimer holes **g**: in the three cases shown, the dielectric constants of the two dimer holes are respectively ε_1_ = 1.21, ε_2_ = 1; (**b**) The dependence of the SMIO state on the difference of the dielectric constants of the two dimer holes **Δε** = ε_1_–ε_2_: in the three cases shown, the center-center distance of the two dimer holes is **g = 0.5d**. In all these results, the angle between the long sides of the holes and the polarization direction of the electric field is ***α*** = 50°.

Due to the properties of interference, the SMIO state is sensitive to the center-center distance *g* of the dimer holes (denoted in the inset of Fig. 3(a)). As the decrement of *g*, the width of the interference region decreases, so the SMIO state shifts to the short wavelength direction (see Fig. 4(a)). We also notice that the transmittance at the SMIO state has a very small change as the decrement of *g*. This illustrates that the robustness of SMIO state does not depend on the distance between the resonant elements. On the other hand, the difference in the electromagnetic property of the dimer holes is also relevant to the appearance of the destructive interference. To demonstrate it properly, we fix ε_2_ as 1 and tune the value of ε_1_. We find that as the decrement of the difference Δε = ε_1_–ε_2_, the transmittance at the SMIO state becomes larger and larger (see Fig. 4(b)), which indicates that the SMIO state becomes weaker and weaker. However, we have to note that even when the difference of the dielectric constant is as low as Δε = 0.01, an obvious transmission dip can be identified, which implies potential sensor applications.

**Figure 5:**
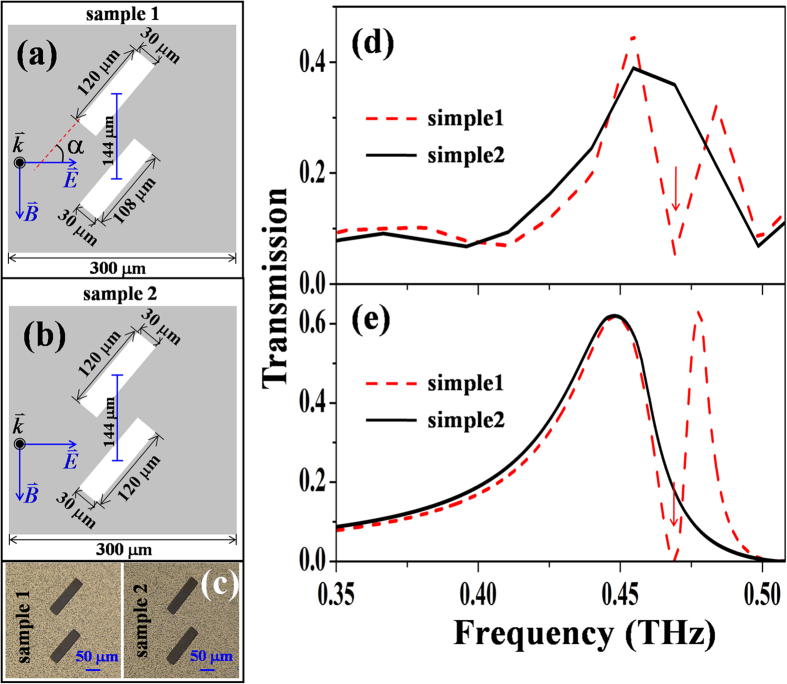
The schematic configurations of the unit cells of two samples: (**a**) the two dimer holes have no difference; (**b**) the two dimer holes have a small difference in their long sides. (**c**) the real image of the two samples: the left one is the sample 1 and the right one is sample 2. The transmission spectra of the two samples: (**d**) shows the measured results; (**e**) shows the calculated results. In (**d**) and (**e**), the red arrows indicate the positions of the SMIO states.

To verify the SMIO state, we fabricate MHA of dimer lattice and measure the transmission spectrum in sub-THz regime. In our samples, the difference between the dimer hole is achieved by shortening the longer sides of one of the dimer holes. The detailed configuration of our sample are shown in Fig. 5(b). As a comparison, we also fabricate a MHA of the identical dimer holes (see Fig. 5(a)). The real images of the two samples are shown in Fig. 5(c). Because MHA is fabricated on a quartz substrate, the wood’s anomaly frequency is given by 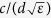 ∼ 0.5 THz. Indeed, for both samples, we observe transmission dips around 0.5 THz. The SMIO state is observed at around 0.47 THz. For the comparison, the calculated transmission spectra are shown in Fig. 5(e) and agree with the experiment data.

## Discussion

Before we make a conclusion, comparisons with the other proposals based on the coupling of localized modes can make our advantages obvious: first, the robustness of SMIO states will not decrease, when the distance between the coupling elements increases, which will happen for the proposals based on localized modes; second, the SMIO proposed here is a bound state formed from the delocalized modes, and thus the resonant frequency of SMIO depends on the distance between two coupling elements (see Fig. 4(a)), which thus provides convenience for tuning; Finally, the SMIO dip is very sensitive to the difference of the two coupling elements (see Fig. 4(b)), which may promise various sensor application.

In conclusion, we have proposed a SMIO state in metal hole array systems, which is demonstrated as an EIT-like phenomena. Different from the existing proposals, the destructive interference in such systems is achieved by the delocalized surface modes. Based on this platform, the interference picture behind the EIT picture is virtualized, and the properties of our proposed system promise various potential applications.

## Method

The theoretical analysis in the work is based on mode expansion formulism^32^,^34^,^35^,^36^ and finite element simulation. The sample fabrication is based on standard photolithography technique. The experimental measurement is based on Terahertz time-domain spectroscopy.

## Additional Information

**How to cite this article**: Xiao, X. *et al.* An Analog of electrically induced transparency via surface delocalized modes. *Sci. Rep.* **5**, 12251; doi: 10.1038/srep12251 (2015).

## Supplementary Material

Supplementary Information
