# Supplementary material for: An Analog of electrically induced transparency via surface delocalized modes
Source: Sci Rep. 2015 Jul 21;5:12251. doi: 10.1038/srep12251 (PMC4508561; doi:10.1038/srep12251)
Supplement: Supplementary Information [file srep12251-s1.pdf]

Supplementary material of “An Analog of electrically induced transparency via surface delocalized modes”

Xiao Xiao[1], Bingpu Zhou[1], Xinke Wang[2], Jingwen He[2], Bo Hou[3], Yan Zhang[2], and Weijia Wen[1,]*

[1] Department of physics, The Hong Kong University of Science and Technology, Clear Water Bay, Kowloon, Hong Kong

[2]Department of Physics, Capital Normal Univeristy, Beijing 100048, China

[3] School of Physical Science and Technology, Suzhou University, No.1 Shizi Street, Soochow, Jiangsu 215006, China

We provide the proofs that the surface modes from the two dimer holes at SMIO frequency are off phase. Moreover, the effect of electromagnetic properties of dimmers on the phenomena is analyzed.

Let us begin from the mode expansion equations:

$$\begin{cases} 2\gamma_{\vec{k}_i,\sigma_i}\left\langle \vec{q}_{l,s},\sigma'',1\middle|\vec{k}_i,\sigma_i\right\rangle \\ -\sum_{m,n,\sigma'}\sum_{l,s,\sigma}\gamma_{\vec{k}_{m,n},\sigma'}\begin{pmatrix}\Sigma_{l,s,\sigma}\left\langle \vec{q}_{l,s},\sigma'',1\middle|\vec{k}_{m,n},\sigma'\right\rangle\left\langle \vec{k}_{m,n},\sigma'\middle|\vec{q}_{l,s},\sigma,1\right\rangle \\ +\Upsilon_{l,s,\sigma}\left\langle \vec{q}_{l,s},\sigma'',1\middle|\vec{k}_{m,n},\sigma'\right\rangle\left\langle \vec{k}_{m,n},\sigma'\middle|\vec{q}_{l,s},\sigma,2\right\rangle\end{pmatrix} = \gamma_{l,s,\sigma''}\left(\dfrac{2\Gamma_{l,s,\sigma''}-\Sigma_{l,s,\sigma''}\left(e^{iq_z^{(l,s)}h}+e^{-iq_z^{(l,s)}h}\right)}{e^{iq_z^{(l,s)}h}-e^{-iq_z^{(l,s)}h}}\right) \\ 2\gamma_{\vec{k}_i,\sigma_i}\left\langle \vec{q}_{l,s},\sigma'',2\middle|\vec{k}_i,\sigma_i\right\rangle \\ -\sum_{m,n,\sigma'}\sum_{l,s,\sigma}\gamma_{\vec{k}_{m,n},\sigma'}\begin{bmatrix}\Sigma_{l,s,\sigma}\left\langle \vec{q}_{l,s},\sigma'',2\middle|\vec{k}_{m,n},\sigma'\right\rangle\left\langle \vec{k}_{m,n},\sigma'\middle|\vec{q}_{l,s},\sigma,1\right\rangle \\ +\Upsilon_{l,s,\sigma}\left\langle \vec{q}_{l,s},\sigma'',2\middle|\vec{k}_{m,n},\sigma'\right\rangle\left\langle \vec{k}_{m,n},\sigma'\middle|\vec{q}_{l,s},\sigma,2\right\rangle\end{bmatrix} = \gamma_{l,s,\sigma''}\left(\dfrac{2\Omega_{l,s,\sigma''}-\left(e^{iq_z^{(l,s)}h}+e^{-iq_z^{(l,s)}h}\right)\Upsilon_{l,s,\sigma''}}{e^{iq_z^{(l,s)}h}-e^{-iq_z^{(l,s)}h}}\right) \\ \gamma_{l,s,\sigma''}\left(\dfrac{\Gamma_{l,s,\sigma''}\left(e^{iq_z^{(l,s)}h}+e^{-iq_z^{(l,s)}h}\right)-2\Sigma_{l,s,\sigma''}}{e^{iq_z^{(l,s)}h}-e^{-iq_z^{(l,s)}h}}\right) \\ =\sum_{m,n,\sigma}\gamma_{m,n,\sigma}\left[\sum_{l,s,\sigma'}\Gamma_{l,s,\sigma'}\left\langle \vec{q}_{l,s},\sigma'',1\middle|\vec{k}_{m,n},\sigma\right\rangle\left\langle \vec{k}_{m,n},\sigma\middle|\vec{q}_{l,s},\sigma',1\right\rangle+\Omega_{l,s,\sigma'}\left\langle \vec{q}_{l,s},\sigma'',1\middle|\vec{k}_{m,n},\sigma\right\rangle\left\langle \vec{k}_{m,n},\sigma\middle|\vec{q}_{l,s},\sigma',2\right\rangle\right] \\ \gamma_{l,s,\sigma''}\left(\dfrac{\Omega_{l,s,\sigma''}\left(e^{iq_z^{(l,s)}h}+e^{-iq_z^{(l,s)}h}\right)-2\Upsilon_{l,s,\sigma''}}{e^{iq_z^{(l,s)}h}-e^{-iq_z^{(l,s)}h}}\right) \\ =\sum_{m,n,\sigma}\gamma_{m,n,\sigma}\left[\sum_{l,s,\sigma'}\Gamma_{l,s,\sigma'}\left\langle \vec{q}_{l,s},\sigma'',2\middle|\vec{k}_{m,n},\sigma\right\rangle\left\langle \vec{k}_{m,n},\sigma\middle|\vec{q}_{l,s},\sigma',1\right\rangle+\Omega_{l,s,\sigma'}\left\langle \vec{q}_{l,s},\sigma'',2\middle|\vec{k}_{m,n},\sigma\right\rangle\left\langle \vec{k}_{m,n},\sigma\middle|\vec{q}_{l,s},\sigma',2\right\rangle\right] \end{cases} \quad (1)$$

In above expression, we have:

$$\begin{cases} \Sigma_{l,s,\sigma} = A^1_{l,s,\sigma} + B^1_{l,s,\sigma} \\ \Gamma_{l,s,\sigma} = A^1_{l,s,\sigma}e^{iq_z^{(l,s)}h} + B^1_{l,s,\sigma}e^{-iq_z^{(l,s)}h} \end{cases} \quad (2)$$

which gives the amplitudes of the electromagnetic field at upper holes in the unit cell for a given mode labeled by l, s, and σ.

$$\begin{cases} \Upsilon_{l,s,\sigma} = A_{l,s,\sigma}^2 + B_{l,s,\sigma}^2 \\ \Omega_{l,s,\sigma} = A_{l,s,\sigma}^2 e^{iq_z^{(l,s)}h} + B_{l,s,\sigma}^2 e^{-iq_z^{(l,s)}h} \end{cases} \tag{3}$$

which gives the amplitudes of the electromagnetic field at lower holes in the unit cell for a given mode labeled by l, s, and σ.

The z-component wave vector for a given waveguide modes is given by $q_z^{(l,s)} = \sqrt{\tilde{\omega}^2 \varepsilon - (l\pi / a)^2 - (s\pi / b)^2}$. The admittances of a mode of given polarization are $\gamma_{\sigma=1} = \frac{k_z}{\tilde{\omega}}$ and $\gamma_{\sigma=2} = \frac{\tilde{\omega}\varepsilon}{k_z}$.

The details of the ket expressions are given in the appendix.

In subwavelength region, we can only consider the fundamental TE mode for the longer sides of the holes. Thus, the equation (1) can be greatly simplified:

$$\begin{cases} 2\gamma_{\vec{k}_i,\sigma_i} \left\langle \vec{q}_{0,1},1,1 \middle| \vec{k}_i,\sigma_i \right\rangle \\ -\sum_{m,n,\sigma'} Y_{\vec{k}_{m,n},\sigma'} \begin{pmatrix} \Sigma_{0,1,1} \left\langle \vec{q}_{0,1},1,1 \middle| \vec{k}_{m,n},\sigma' \right\rangle \left\langle \vec{k}_{m,n},\sigma' \middle| \vec{q}_{0,1},1,1 \right\rangle \\ +\Upsilon_{0,1,1} \left\langle \vec{q}_{0,1},1,1 \middle| \vec{k}_{m,n},\sigma' \right\rangle \left\langle \vec{k}_{m,n},\sigma' \middle| \vec{q}_{0,1},1,2 \right\rangle \end{pmatrix} = \gamma_{0,1,1} \left(\frac{2\Gamma_{0,1,1} - \Sigma_{0,1,1}\left(e^{iq_z^{(0,1)}h} + e^{-iq_z^{(0,1)}h}\right)}{e^{iq_z^{(0,1)}h} - e^{-iq_z^{(0,1)}h}} \right) \\ 2\gamma_{\vec{k}_i,\sigma_i} \left\langle \vec{q}_{0,1},1,2 \middle| \vec{k}_i,\sigma_i \right\rangle \\ -\sum_{m,\sigma'} Y_{\vec{k}_{m,n},\sigma'} \begin{bmatrix} \Sigma_{0,1,1} \left\langle \vec{q}_{0,1},1,2 \middle| \vec{k}_{m,n},\sigma' \right\rangle \left\langle \vec{k}_{m,n},\sigma' \middle| \vec{q}_{0,1},1,1 \right\rangle \\ +\Upsilon_{0,1,1} \left\langle \vec{q}_{0,1},1,2 \middle| \vec{k}_{m,n},\sigma' \right\rangle \left\langle \vec{k}_{m,n},\sigma' \middle| \vec{q}_{0,1},1,2 \right\rangle \end{bmatrix} = \gamma_{0,1,1} \left(\frac{2\Omega_{0,1,1} - \left(e^{iq_z^{(0,1)}h} + e^{-iq_z^{(0,1)}h}\right)\Upsilon_{0,1,1}}{e^{iq_z^{(0,1)}h} - e^{-iq_z^{(0,1)}h}} \right) \\ \gamma_{0,1,1} \left(\frac{\Gamma_{0,1,1}\left(e^{iq_z^{(0,1)}h} + e^{-iq_z^{(0,1)}h}\right) - 2\Sigma_{0,1,1}}{e^{iq_z^{(0,1)}h} - e^{-iq_z^{(0,1)}h}} \right) = \sum_{m,n,\sigma} Y_{\vec{k}_{m,n},\sigma} \begin{bmatrix} \Gamma_{0,1,1} \left\langle \vec{q}_{0,1},1,1 \middle| \vec{k}_{m,n},\sigma \right\rangle \left\langle \vec{k}_{m,n},\sigma \middle| \vec{q}_{0,1},1,1 \right\rangle \\ +\Omega_{0,1,1} \left\langle \vec{q}_{0,1},1,1 \middle| \vec{k}_{m,n},\sigma \right\rangle \left\langle \vec{k}_{m,n},\sigma \middle| \vec{q}_{0,1},1,2 \right\rangle \end{bmatrix} \\ \gamma_{0,1,1} \left(\frac{\Omega_{0,1,1}\left(e^{iq_z^{(0,1)}h} + e^{-iq_z^{(0,1)}h}\right) - 2\Upsilon_{0,1,1}}{e^{iq_z^{(0,1)}h} - e^{-iq_z^{(0,1)}h}} \right) = \sum_{m,n,\sigma} Y_{\vec{k}_{m,n},\sigma} \begin{bmatrix} \Gamma_{0,1,1} \left\langle \vec{q}_{0,1},1,2 \middle| \vec{k}_{m,n},\sigma \right\rangle \left\langle \vec{k}_{m,n},\sigma \middle| \vec{q}_{0,1},1,1 \right\rangle \\ +\Omega_{0,1,1} \left\langle \vec{q}_{0,1},1,2 \middle| \vec{k}_{m,n},\sigma \right\rangle \left\langle \vec{k}_{m,n},\sigma \middle| \vec{q}_{0,1},1,2 \right\rangle \end{bmatrix} \end{cases} \tag{4}$$

Then we compare the result of the full calculation (2 waveguide modes is enough to make the result converge.) and the single waveguide mode result.

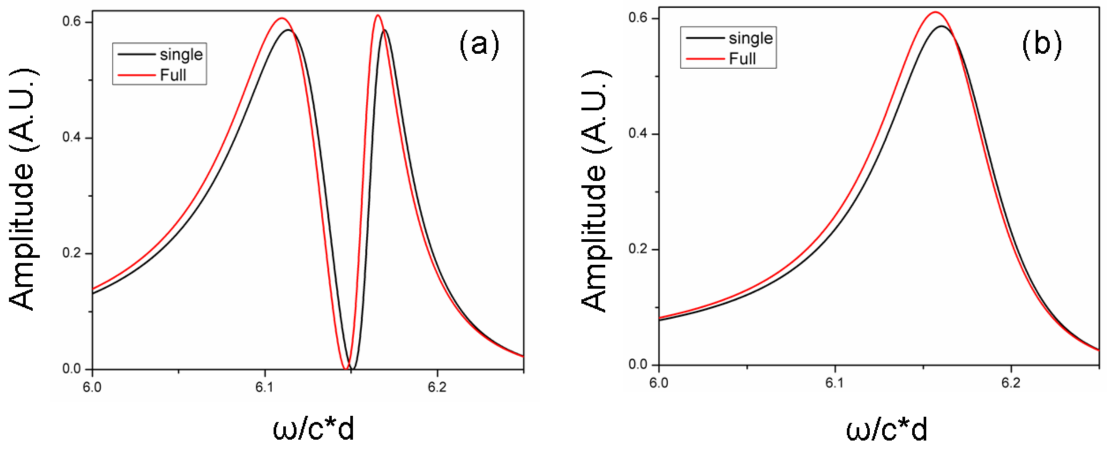

Fig.1 Transmission spectra: single mode VS Full calculation

It can be seen that the single mode approximation does not derivate very much from the full calculation, and all features of the transmission spectra have been captured by the single mode approximation.

By defining the following correlation functions:

$$\frac{2\gamma_{0,1,1}}{\left(e^{iq_z^{(0,1)}h}-e^{-iq_z^{(0,1)}h}\right)}=G_{\text{layer},1} \tag{5}$$

$$\frac{2\gamma_{0,1,1}}{\left(e^{iq_{z1}^{(0,1)}h}-e^{-iq_{z1}^{(0,1)}h}\right)}=G_{\text{layer},2} \tag{6}$$

$$\begin{cases}\left[\sum_{m,n,\sigma'} Y_{\vec{k}_{m,n},\sigma}\left\langle \vec{q}_{0,1},1,1\middle|\vec{k}_{m,n},\sigma\right\rangle\left\langle \vec{k}_{m,n},\sigma\middle|\vec{q}_{0,1},1,1\right\rangle\right]=G_{\text{surface}}^{11}\\ \left[\sum_{m,n,\sigma'} Y_{\vec{k}_{m,n},\sigma}\left\langle \vec{q}_{0,1},1,1\middle|\vec{k}_{m,n},\sigma\right\rangle\left\langle \vec{k}_{m,n},\sigma\middle|\vec{q}_{0,1},1,2\right\rangle\right]=G_{\text{surface}}^{12}\\ \left[\sum_{m,n,\sigma'} Y_{\vec{k}_{m,n},\sigma}\left\langle \vec{q}_{0,1},1,2\middle|\vec{k}_{m,n},\sigma\right\rangle\left\langle \vec{k}_{m,n},\sigma\middle|\vec{q}_{0,1},1,1\right\rangle\right]=G_{\text{surface}}^{21}\\ \left[\sum_{m,n,\sigma'} Y_{\vec{k}_{m,n},\sigma}\left\langle \vec{q}_{0,1},1,2\middle|\vec{k}_{m,n},\sigma\right\rangle\left\langle \vec{k}_{m,n},\sigma\middle|\vec{q}_{0,1},1,2\right\rangle\right]=G_{\text{surface}}^{22}\end{cases} \tag{7}$$

$$\frac{\gamma_{0,1,1}\left(e^{iq_z^{(0,1)}h}+e^{-iq_z^{(0,1)}h}\right)}{\left(e^{iq_z^{(0,1)}h}-e^{-iq_z^{(0,1)}h}\right)}=\alpha_{WGM,1}^{-1} \tag{8}$$

$$\frac{\gamma_{0,1,1}\left(e^{iq_{z1}^{(0,1)}h}+e^{-iq_{z1}^{(0,1)}h}\right)}{\left(e^{iq_{z1}^{(0,1)}h}-e^{-iq_{z1}^{(0,1)}h}\right)}=\alpha_{WGM,2}^{-1} \tag{9}$$

$$\begin{cases} 2\gamma_{\vec{k}_i,\sigma_i}\left\langle \vec{q}_{0,1},1,1\middle|\vec{k}_i,\sigma_i\right\rangle = I_1 \\ 2\gamma_{\vec{k}_i,\sigma_i}\left\langle \vec{q}_{0,1},1,2\middle|\vec{k}_i,\sigma_i\right\rangle = I_2 \end{cases} \quad (10)$$

The equation (4) can be re-written as:

$$\begin{cases} \left(G_{\text{surface}}^{11} - \alpha_{WGM,1}^{-1}\right)\Sigma_{0,1,1} + G_{\text{surface}}^{12}\Upsilon_{0,1,1} + G_{\text{layer}}^{1}\Gamma_{0,1,1} = I_1 \\ \left(G_{\text{surface}}^{22} - \alpha_{WGM,2}^{-1}\right)\Upsilon_{0,1,1} + G_{\text{surface}}^{21}\Sigma_{0,1,1} + G_{\text{layer}}^{2}\Omega_{0,1,1} = I_2 \\ \left(G_{\text{surface}}^{11} - \alpha_{WGM,1}^{-1}\right)\Gamma_{0,1,1} + G_{\text{surface}}^{12}\Omega_{0,1,1} + G_{\text{layer}}^{1}\Sigma_{0,1,1} = 0 \\ \left(G_{\text{surface}}^{22} - \alpha_{WGM,2}^{-1}\right)\Omega_{0,1,1} + G_{\text{surface}}^{21}\Gamma_{0,1,1} + G_{\text{layer}}^{2}\Upsilon_{0,1,1} = 0 \end{cases} \quad (11)$$

From the expressions of the ket, one can find that (also proved by numerical calculation):

$$\begin{cases} G_{\text{surface}}^{11} = G_{\text{surface}}^{22} = G_{lattice} \\ G_{\text{surface}}^{12} = G_{\text{surface}}^{21} = G_{cell} \end{cases} \quad (12)$$

$$\begin{cases} G_{lattice} - \alpha_{WGM,1}^{-1} = G_1 \\ G_{lattice} - \alpha_{WGM,2}^{-1} = G_2 \end{cases} \quad (13)$$

Then the equation (11) becomes:

$$\begin{cases} G_1\Sigma_{0,1,1} + G_{cell}\Upsilon_{0,1,1} + G_{\text{layer},1}\Gamma_{0,1,1} = I_1 \\ G_2\Upsilon_{0,1,1} + G_{cell}\Sigma_{0,1,1} + G_{\text{layer},2}\Omega_{0,1,1} = I_2 \\ G_1\Gamma_{0,1,1} + G_{cell}\Omega_{0,1,1} + G_{\text{layer},1}\Sigma_{0,1,1} = 0 \\ G_2\Omega_{0,1,1} + G_{cell}\Gamma_{0,1,1} + G_{\text{layer},2}\Upsilon_{0,1,1} = 0 \end{cases} \quad (14)$$

One can solve out the expression for the field amplitudes of waveguide modes at the output interface analytically, and they are:

$$\Gamma_{0,1,1} = -\frac{G_{\text{layer},1}G_2{}^2 I_1 + \left(G_{cell}\right)^2 G_{\text{layer},2} I_1 - G_{\text{layer},1}G_{\text{layer},2}{}^2 I_1 - G_{cell}G_{\text{layer},1}G_2 I_2 - G_1 G_{cell}G_{\text{layer},2} I_2}{\left(G_{cell}\right)^4 - 2G_1\left(G_{cell}\right)^2 G_2 + G_1{}^2 G_2{}^2 - G_{\text{layer},1}{}^2 G_2{}^2 - 2G_{\text{layer},1}\left(G_{cell}\right)^2 G_{\text{layer},2} - G_1{}^2 G_{\text{layer},2}{}^2 + G_{\text{layer},1}{}^2 G_{\text{layer},2}{}^2} \quad (15)$$

$$\Omega_{0,1,1} = \frac{-G_{cell}G_{\text{layer},1}G_2 I_1 - G_1 G_{cell}G_{\text{layer},2} I_1 + \left(G_{cell}\right)^2 G_{\text{layer},1} I_2 + G_1{}^2 G_{\text{layer},2} I_2 - G_{\text{layer},1}{}^2 G_{\text{layer},2} I_2}{\left(G_{cell}\right)^4 - 2G_1\left(G_{cell}\right)^2 G_2 + G_1{}^2 G_2{}^2 - G_{\text{layer},1}{}^2 G_2{}^2 - 2G_{\text{layer},1}\left(G_{cell}\right)^2 G_{\text{layer},2} - G_1{}^2 G_{\text{layer},2}{}^2 + G_{\text{layer},1}{}^2 G_{\text{layer},2}{}^2} \quad (16)$$

Then the transmission coefficient is gotten by:

$$T = \left|\Gamma_{0,1,1}S_0\sin\theta + \Omega_{0,1,1}S_0\sin\varphi\right|^2 + \left|\Gamma_{0,1,1}S_0\cos\theta + \Omega_{0,1,1}S_0\cos\varphi\right|^2 \quad (17)$$

Where φ (θ) is angle of the upper (lower) hole tilting from y axis. Since $\varphi = \theta$, we can further simplify (54) as:

$$T = \left|\Gamma_{0,1,1} + \Omega_{0,1,1}\right|^2 \left|S_0\right|^2 \sin^2\theta + \left|\Gamma_{0,1,1} + \Omega_{0,1,1}\right|^2 \left|S_0\right|^2 \cos^2\theta = \left|\Gamma_{0,1,1} + \Omega_{0,1,1}\right|^2 \left|S_0\right|^2 \quad (18)$$

Since the shape of the hole are the same, $I_1 = I_2 = I$, which can further simplify the expressions.

A. The case without difference in dielectric constants:

To feeling the power of the results above, we firstly consider the case that the two holes in the unit cell fills with nothing. Thus, one can have $G_1 = G_2$ and $G_{\text{layer},1} = G_{\text{layer},2}$. Consequently,

$$Numerator\left(\Gamma_{0,1,1}+\Omega_{0,1,1}\right)=\begin{bmatrix} -2\left(G_{cell}G_{\text{layer},1}G_2+G_1G_{cell}G_{\text{layer},2}\right)+\left(G_{\text{layer},1}G_2{}^2+G_1{}^2G_{\text{layer},2}\right) \\ +\left[\left(G_{cell}\right)^2-G_{\text{layer},1}G_{\text{layer},2}\right]\left(G_{\text{layer},1}+G_{\text{layer},2}\right) \end{bmatrix} I$$

$$=G_{\text{layer},1}\left[-4G_{cell}G_1+2G_1{}^2+2\left(\left(G_{cell}\right)^2-G_{\text{layer},1}{}^2\right)\right]I=2G_{\text{layer},1}\left[\left(G_{cell}-G_1\right)^2-G_{\text{layer},1}{}^2\right]I \tag{19.a}$$

$$denominator\left(\Gamma_{0,1,1}+\Omega_{0,1,1}\right)=\begin{Bmatrix} \left(G_{cell}\right)^4-2G_1\left(G_{cell}\right)^2G_2+G_1{}^2G_2{}^2-G_{\text{layer},1}{}^2G_2{}^2 \\ -2G_{\text{layer},1}\left(G_{cell}\right)^2G_{\text{layer},2}-G_1{}^2G_{\text{layer},2}{}^2+G_{\text{layer},1}{}^2G_{\text{layer},2}{}^2 \end{Bmatrix}$$

$$=\begin{Bmatrix} \left(G_{cell}\right)^4-2G_1{}^2\left(G_{cell}\right)^2+G_1{}^4-2G_{\text{layer},1}{}^2G_1{}^2 \\ -2\left(G_{cell}\right)^2G_{\text{layer},1}{}^2+G_{\text{layer},1}{}^4 \end{Bmatrix}$$

$$=\left\{\left(G_{cell}{}^2-G_1{}^2\right)^2-2G_{\text{layer},1}{}^2\left(G_{cell}{}^2+G_1{}^2\right)+G_{\text{layer},1}{}^4\right\} \tag{19.b}$$

$$=\left\{\left(G_{cell}-G_1\right)^2\left(G_{cell}+G_1\right)^2-2G_{\text{layer},1}{}^2\left(G_{cell}{}^2+G_1{}^2\right)+G_{\text{layer},1}{}^4\right\}$$

$$=\left\{\left[\left(G_{cell}-G_1\right)^2-G_{\text{layer},1}{}^2\right]\left[\left(G_{cell}+G_1\right)^2-G_{\text{layer},1}{}^2\right]\right\}$$

As a result, we have a very simple expression for the transmission:

$$T=\left|\frac{2G_{\text{layer},1}I}{\left[\left(G_{cell}+G_1\right)^2-G_{\text{layer},1}{}^2\right]}\right|^2\left|S_0\right|^2 \tag{20}$$

where $S_0=\left\langle \vec{q}_{0,1},1,i=1,2\middle|\vec{k}_{0,0},\sigma'=2\right\rangle$. The correction of (20) can be seen by comparing with numerical solution.

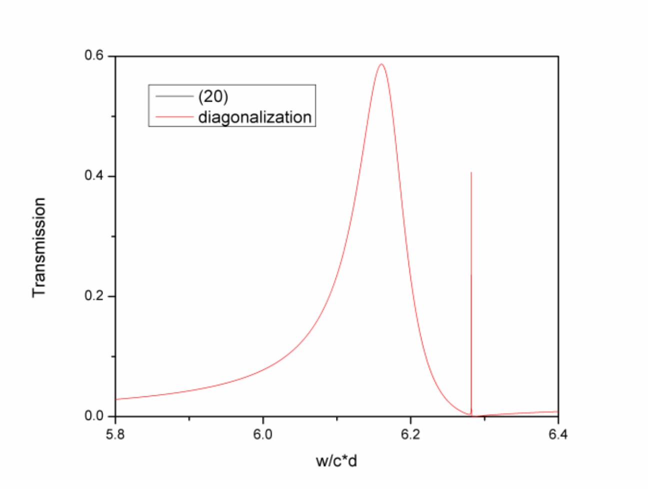

Fig.2 Transmission spectra for the case of no difference in dielectric constants

One can observe that one term in the denominator cancels the same term in the numerator. We can expect that when the small dielectric difference is taken into account, the term would not be cancelled, because of the different deviations in denominator and numerator. On the other hand, we can also expect that the deviations should be small, since the dielectric difference is small. Therefore, it would be useful for us to plot $\left[\left(G_{cell}-G_1\right)^2-G_{\text{layer},1}{}^2\right]$.

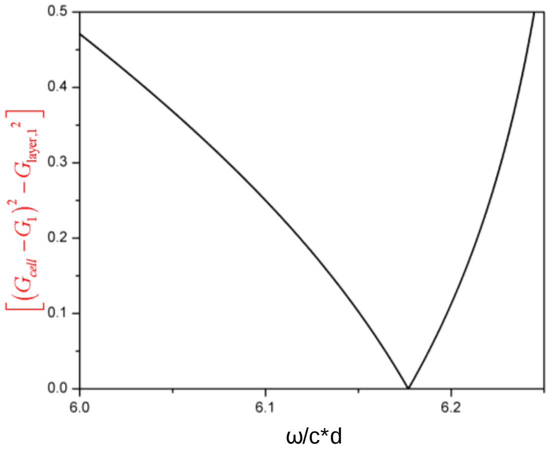

Fig. 3 the plot of $\left[\left(G_{cell}-G_1\right)^2-G_{\text{layer},1}{}^2\right]$

One can expect that the dielectric difference would shift the 0 points of the term. The other term $2G_{\text{layer},1}I \approx 2G_{\text{layer},2}I$ is roughly a constant in the region of interest ([6.0, 6.25]). The change of the 0 points of the term $\left[\left(G_{cell}-G_1\right)^2-G_{\text{layer},1}{}^2\right]$ tunes the 0 points of the Fano profile (For hole array system on single plate, the transmission almost results from Fano resonance.), and thus it tunes the Fano factor $q_F = 2\left(\omega_r-\omega_0\right)/\Gamma$, where ω_r is the resonant frequency of the structure, ω_0 is the 0 point of the transmission spectrum, and Γ is the bandwidth of the resonance. For the hole array system on the single plate, the resonance can be regarded from the formation of standing wave of the surface modes. Thus, the resonant frequency ω_r would not change, if the lattice is preserved with no change.

II. The case with difference in dielectric constants:

From (15) and (16), we observe that the denominators of the field amplitudes from the upper and lower holes in the unit cell are the same. Let us firstly check the behavior of it and rule out the effect from the denominator. We will plot the denominator in the Fig. 4.

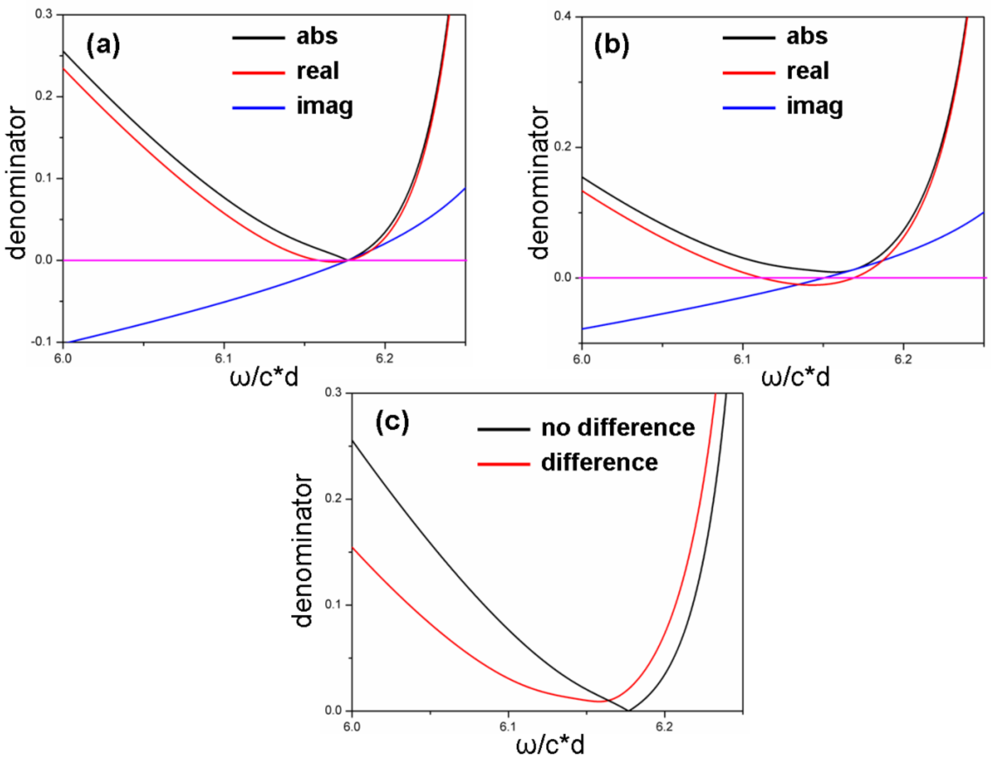

Fig.4 The plot of the denominators for the two holes with the same (a) and different (b) dielectric constant; (c) the comparison of the absolute value for the same and different dielectric constants in two holes.

It can be seen from Fig. 4 that the difference in the dielectric constants shifts slightly the real and imaginary part respectively (see (a) and (b)) so that the 0 point of the denominator is removed (see (c)).

Thus, we can say that the off-phase should result from the numerator. We treat the field amplitudes of the two holes respectively:

$$\begin{cases} Num(\Gamma_{0,1,1}) = \left(G_{\text{layer},1} G_2^{\;2} + (G_{cell})^2 G_{\text{layer},2} - G_{\text{layer},1} G_{\text{layer},2}^{\;\;2} - G_{cell} G_{\text{layer},1} G_2 - G_1 G_{cell} G_{\text{layer},2}\right) I \\ Num(\Omega_{0,1,1}) = \left(-G_{cell} G_{\text{layer},1} G_2 - G_1 G_{cell} G_{\text{layer},2} + (G_{cell})^2 G_{\text{layer},1} + G_1^{\;2} G_{\text{layer},2} - G_{\text{layer},1}^{\;\;2} G_{\text{layer},2}\right) I \end{cases} \tag{21}$$

The numerical calculation shows that in the region of interest:

$$\begin{cases} |G_{cell}| \sim |G_1| \approx |G_2| \approx |G_{layer,1}| \approx |G_{layer,2}| \\ |G_1| \approx |G_{layer,1}| \sim |G_{layer,1} - G_{layer,2}| \approx |G_1 - G_2| \end{cases} \tag{22}$$

We define:

$$\begin{cases} G_{\text{layer},2} - G_{\text{layer},1} = \Delta G_{\text{layer}} \\ G_2 - G_1 = \Delta G \end{cases} \tag{23}$$

For the case without dielectric constant difference, $\Delta G_{\text{layer}} = 0$ and $\Delta G = 0$.

We identify $G_{cell} \approx \Delta G_{\text{layer}} \approx \Delta G$ are in the same order. Then we limit ourselves to the order $O(G_{cell})$. Thus, (21) can be written as:

$$Num(\Gamma_{0,1,1}) = \begin{pmatrix} G_{\mathrm{layer},1}\left(G_1+\Delta G\right)^2 + \left(G_{cell}\right)^2\left(G_{\mathrm{layer},1}+\Delta G_{\mathrm{layer}}\right) - G_{\mathrm{layer},1}\left(G_{\mathrm{layer},1}+\Delta G_{\mathrm{layer}}\right)^2 \\ -G_{cell}G_{\mathrm{layer},1}\left(G_1+\Delta G\right) - G_1 G_{cell}\left(G_{\mathrm{layer},1}+\Delta G_{\mathrm{layer}}\right) \end{pmatrix} I$$

$$= \begin{pmatrix} G_{\mathrm{layer},1}\left(G_1{}^2+2\Delta G G_1+\Delta G^2\right) + \left(G_{cell}\right)^2\left(G_{\mathrm{layer},1}+\Delta G_{\mathrm{layer}}\right) \\ -G_{\mathrm{layer},1}\left(G_{\mathrm{layer},1}{}^2+2\Delta G_{\mathrm{layer}}G_{\mathrm{layer},1}+\Delta G_{\mathrm{layer}}{}^2\right) \\ -G_{cell}G_{\mathrm{layer},1}\left(G_1+\Delta G\right) - G_1 G_{cell}\left(G_{\mathrm{layer},1}+\Delta G_{\mathrm{layer}}\right) \end{pmatrix} I$$

$$\approx \begin{pmatrix} G_{\mathrm{layer},1}G_1{}^2 + 2\Delta G G_1 G_{\mathrm{layer},1} + \left(G_{cell}\right)^2 G_{\mathrm{layer},1} \\ -G_{\mathrm{layer},1}{}^3 - 2\Delta G_{\mathrm{layer}}G_{\mathrm{layer},1}{}^2 - 2G_{cell}G_{\mathrm{layer},1} \end{pmatrix} I = \begin{pmatrix} G_{\mathrm{layer},1}G_1{}^2 + \left(G_{cell}\right)^2 G_{\mathrm{layer},1} - G_{\mathrm{layer},1}{}^3 - 2G_{cell}G_{\mathrm{layer},1}G_1 \\ -2\Delta G_{\mathrm{layer}}G_{\mathrm{layer},1}{}^2 + 2\Delta G G_1 G_{\mathrm{layer},1} \end{pmatrix} I \tag{24}$$

The upper row in the last equity is exactly the case without difference in dielectric constant. The lower row gives the shift. We do the same thing for the other term:

$$Num(\Omega_{0,1,1}) = \left(-G_{cell}G_{\mathrm{layer},1}G_2 - G_1 G_{cell}G_{\mathrm{layer},2} + \left(G_{cell}\right)^2 G_{\mathrm{layer},1} + G_1{}^2 G_{\mathrm{layer},2} - G_{\mathrm{layer},1}{}^2 G_{\mathrm{layer},2}\right) I$$

$$= \begin{pmatrix} -2G_{cell}G_{\mathrm{layer},1}G_1 + \left(G_{cell}\right)^2 G_{\mathrm{layer},1} + G_1{}^2 G_{\mathrm{layer},1} - G_{\mathrm{layer},1}{}^3 \\ +G_1{}^2\Delta G_{\mathrm{layer}} - G_{\mathrm{layer},1}{}^2\Delta G_{\mathrm{layer}} \end{pmatrix} I \tag{25}$$

Then we plot the numerators of the field amplitude at the upper hole ($\Gamma_{0,1,1}$) and that at the lower hole ($\Omega_{0,1,1}$).

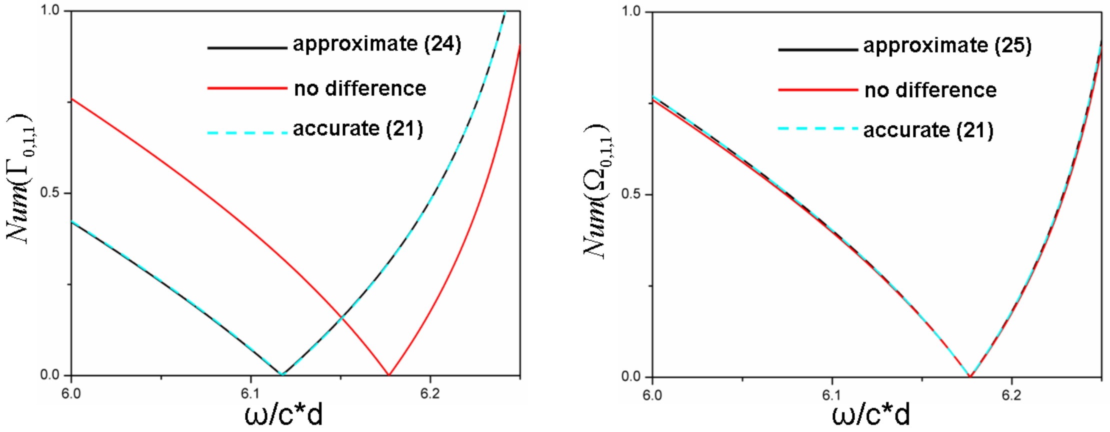

Fig. 5 the plots of the absolute values of the numerators of $\Gamma_{0,1,1}$ (a) and $\Omega_{0,1,1}$ (b)

One can find that the 0 point of $\Gamma_{0,1,1}$ shifts to a lower frequency, and the 0 point of $\Omega_{0,1,1}$ has nearly no changes. As we have known for the no-difference case, the transmission profile is Fano profile, which means $\omega_r - \omega_0 < 0$. Therefore, we may expect that the contribution from $\Gamma_{0,1,1}$ may give an anti-Fano profile $\omega_r - \omega_0 > 0$, which ω_0 becomes smaller. Then we check it, One trivial way to see it is to plot $\left|\Gamma_{0,1,1}\right| / \max(\Gamma_{0,1,1})$ and $\left|\Omega_{0,1,1}\right| / \max(\Omega_{0,1,1})$.

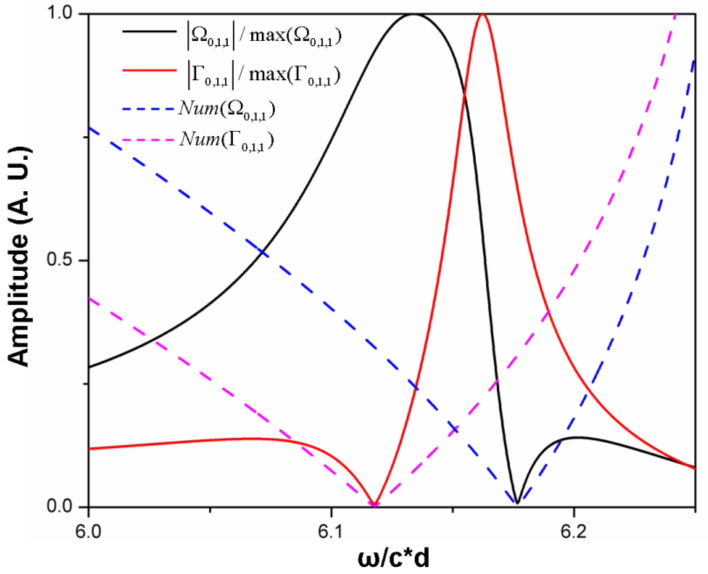

Fig. 6 the plot of $|\Gamma_{0,1,1}| / \max(\Gamma_{0,1,1})$ and $|\Omega_{0,1,1}| / \max(\Omega_{0,1,1})$ with comparison to the absolute values of $Num(\Gamma_{0,1,1})$ and $Num(\Omega_{0,1,1})$.

Indeed the shift of the 0 points of the numerator induces the change of the transmission profile from Fano to anti-Fano. The amplitude of $\Gamma_{0,1,1}$ is always opposite to that of $\Omega_{0,1,1}$ in the frequency region between the 0 points of the two numerators.

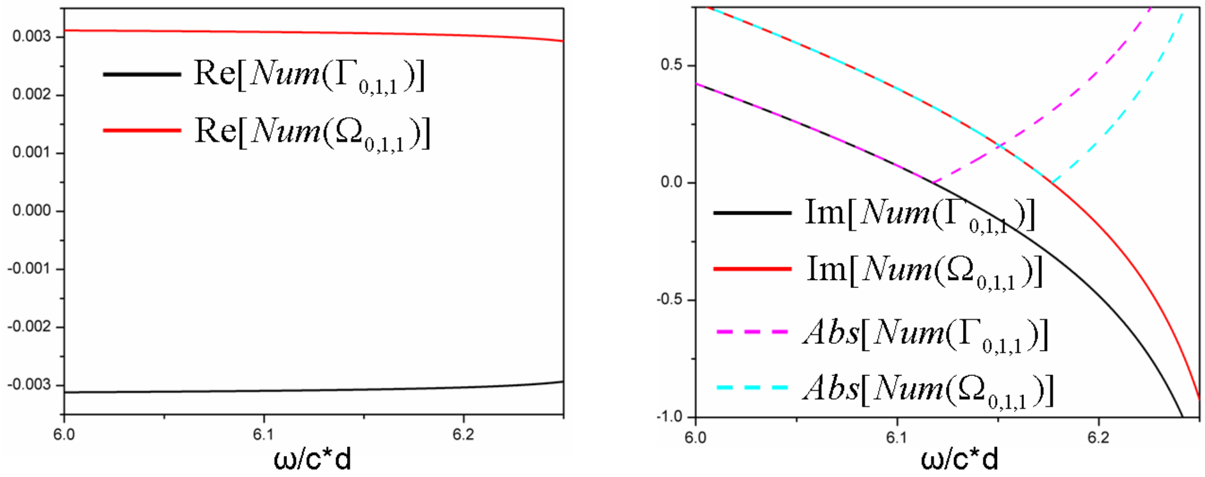

Fig. 7 the plot of the absolute values, real parts and imaginary parts of the numerators of $\Gamma_{0,1,1}$ and $\Omega_{0,1,1}$

From Fig. 7, It can be seen that the real parts of them are always in opposite signs, and the imaginary parts are in opposite signs in the region between two 0 points.

Then we can expect that the cross point of the two absolute values should be identical to the SMIO frequency. To see it, we will plot them in Fig. 8.

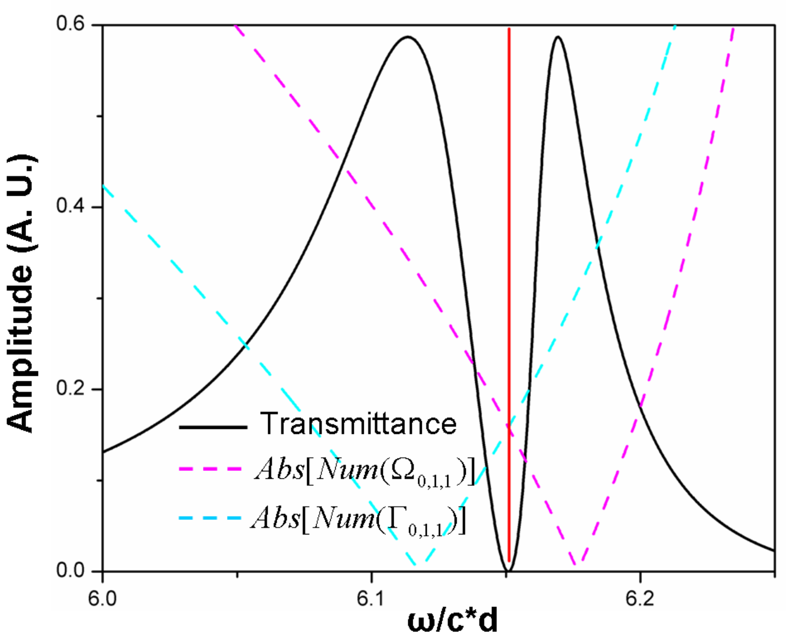

Fig. 8 the plot of the transmission and the absolute values of the numerators; the red line denotes that the cross point of the two absolute values is identical to the SMIO frequency.

In conclusion, the SMIO phenomenon can be viewed in the following way: the introduction of the difference in the dielectric constants of helps the 0 points of the field amplitudes at the two holes to join into the physical picture. As a consequence, the dielectric constant difference shifts the 0 points of the field amplitudes of the two holes from each other, and thus one of them gives a Fano transmission profile and the other gives anti-Fano. In the frequency region between the two 0 points, the amplitudes of the two fields are opposite in signs, which provides the cancellation and gives the transmission dip. All these eigenmodes are bounded on the metal surface and thus are surface modes. Therefore, the transmission dip is indeed due to the interaction between two surface modes launching out from the two dimer holes, which are different in phase.

III. The effect of angle and dielectric constant difference:

In the part, we will check whether the understanding above can be applied to different tilting angles and different dielectric constant differences. We will plot the absolute value of the numerators of the field amplitudes at the two holes ($\Gamma_{0,1,1}$ and $\Omega_{0,1,1}$), and comparing the results with Fig. 3 of the manuscript. We will find the position of SMIO fits well with the 0 points of the numerators. The results are summarized in Fig. 9 below.

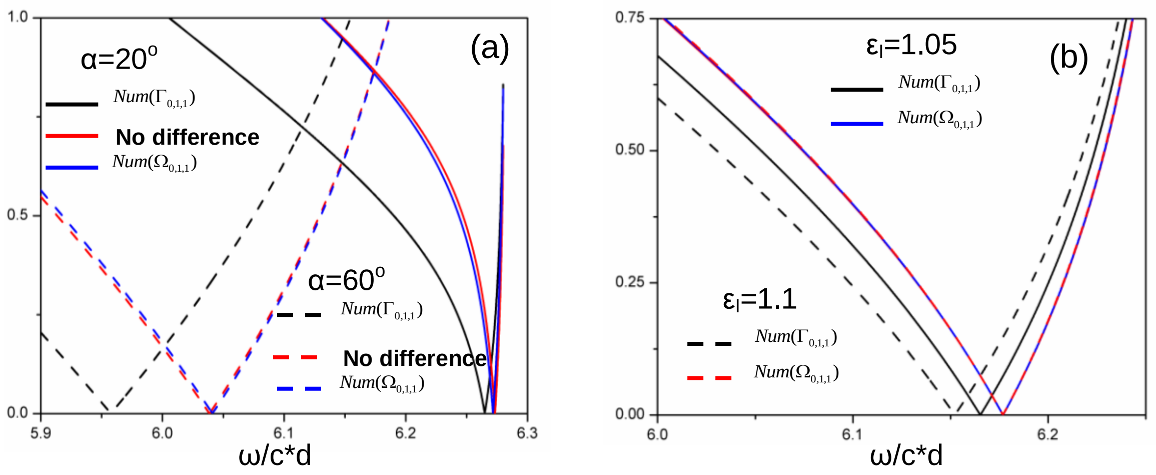

Fig. 9 The plot of the absolute value of the numerators of $\Gamma_{0,1,1}$ and $\Omega_{0,1,1}$
